# Modelling the saturation flow rate for continuous flow intersections based on field collected data

**DOI:** 10.1371/journal.pone.0236922

**Published:** 2020-08-05

**Authors:** Xing Gao, Jing Zhao, Meng Wang

**Affiliations:** 1 Department of Traffic Engineering, University of Shanghai for Science and Technology, Shanghai, China; 2 Department of Transport & Planning, Delft University of Technology, Delft, Netherlands; Tongii University, CHINA

## Abstract

The continuous flow intersection [CFI) is an unconventional intersection design which has been used in many cities all over the world. However, as an important parameter for the geometric design, signal timing, and operation evaluation, the saturation flow rate at CFI has not been carefully examined. In this paper, a saturation flow rate adjustment model for CFI intersections is established based on field data under the calculation framework of the HCM2016. Four movements related with CFI, namely the left-turn at the CFI approach, through movement at the CFI approach, left-turn at the pre-signal, and exit movement at the pre-signal, are discussed. The proposed model is validated and the relative error is less than 10%. The treatments to mitigate the negative impact on the saturation flow rate at the CFI are also recommended. The results show the saturation flow rate of the first three movements are decreased due to the CFI control. For the left-turn, through movement, and left-turn at the pre-signal, the degree of the reduction of the saturation flow rate are mainly related to the heavy vehicle ratio, the proportion of lane changing vehicles, and the length of displaced left-turn lanes, respectively.

## Introduction

The saturation flow rate is the maximum capacity of continuous traffic flow at intersections during the green time in an hour, which is a vital parameter to calculate vehicle delay and queue length at signalized intersections and a basic parameter to determine the green ratio. Therefore, analysing saturation flow rate is of great significance to the geometric design, signal timing, and operation evaluation of signalized intersections. Many works have been done to establish the adjustment factors of signalized intersection capacity. For some influencing factors with common cognition, such as the lane width, heavy vehicles, grade, parking, bus blockage, area type, lane utilization, right turns, left turns, and pedestrians and bicycles, have been subordinated to the widely used highway capacity manuals, such as HCM2016 [1]. Other adjustment factors, such as short-lane [2, 3], driving behaviour [4], red light camera [5], green signal countdown device [6], work zone [7], Electric bicycles [8], and guideline markings [9] have also been discussed.

**Fig 1:**
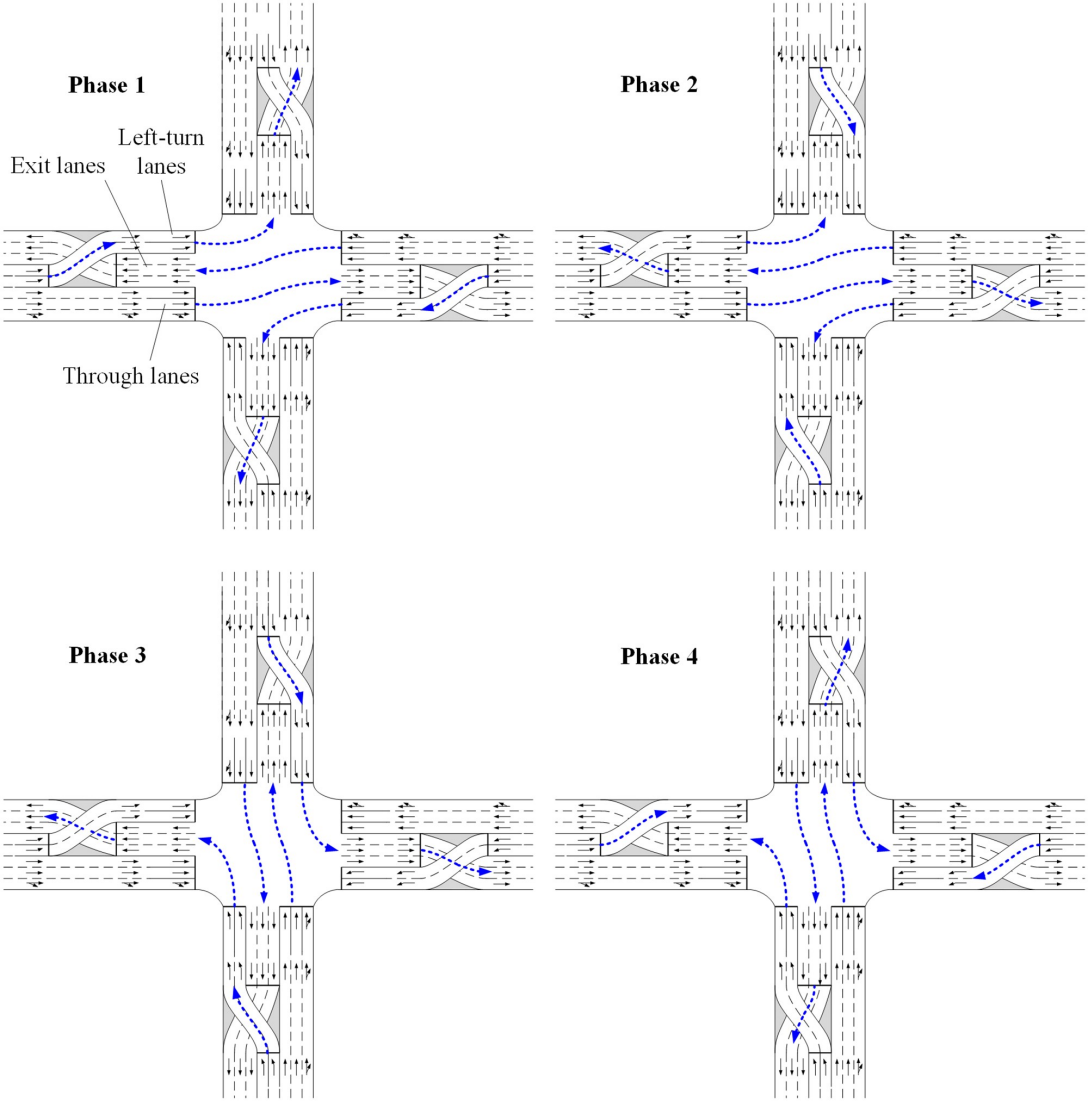
Phase plan and movements at CFI.

With the increasing traffic demand in recent years, various unconventional intersection designs have been investigated by traffic engineers to reduce congestion and improve operational efficiency in the urban road network [10–14]. One of them is the continuous flow intersection (CFI), which is the focus of this study. At continuous flow intersections, the pre-signals are set at the upstream of the main signals and left-turn lanes are moved to the left of the exit-lanes, therefore the conflicts between the left-turn vehicles and opposing-through vehicles at main signals can be resolved at the upstream pre-signals in advance and then the left-turn and through movement of the two opposing legs can run simultaneously without conflict. If such design is used on all the four legs, the main signals will run in two phases. As shown in Fig 1, there are four phases with the combination of main signal and pre-signal at CFI. But they are both in two phases at the main signal and pre-signal alone. This is why the CFI design can enhance the capacity of the intersection. The movements of each phase shown in the figure are helpful to understand how the signal of the CFI design operates more clearly. This is the reason why the continuous flow intersections can enhance the intersection capacity. The continuous flow intersection was first proposed by Mier in 1987 [15]. After more than 30 years of development, the CFI has been taken into application in many countries including USA, Mexico, UK, Germany, Australia, and China [16, 17].

The advantages of CFI design are mainly embodied in setting sub intersections at the upstream of the main intersection, which can divide left-turn vehicles in advance and relieve conflicts between left-turn and opposing-through vehicles at main intersections by pre-signals [18]. In the development of the CFI, studies mainly contain three aspects: geometric design, signal control, and operational safety. Tanwanichkul et al. [19] analysed the distance requirement between main intersection and sub intersection under different traffic conditions by simulation. Hughes et al. [17] gave a series of detailed suggestions on left-turn lane length, pre-signal intersection width, turning radius and other minor dimensions. Tarko et al. [20] made signal timing for the main and pre-signals at continuous flow intersections by using Synchro software. Chang et al. [16] established queue and delay estimation models by simulating a large number of statistical data. Coates et al. [21] proposed geometric design and signal timing for improving pedestrian crossing efficiency. The signal could dynamically choose which phases to use to reduce pedestrian waiting time and existing queue length as far as possible. Zhao et al. [22] proposed an alternative design for left-turn bicycles to eliminate the conflict between left-turn bicycles and through vehicles at CFI intersections. The results show that the vehicular capacity can be improved and the delay of bicycles can be reduced in the case of high traffic demand.

However, the adjustment of the saturation flow rate for unconventional intersection has not been included in the highway capacity manual, such as HCM2016 [1]. Although many studies were carried out about the optimal design of the CFI and the problems of unusual driving behaviour when drivers meet continuous flow intersections was recognized in previous studies, engineers cannot adjust saturation flow rate of continuous flow intersections specifically. This leads to inaccurate operation evaluation and inappropriate design for unconventional intersection.

In order to deal with the deficiency, the objective of this study is to develop a saturation flow rate adjustment model for CFI intersections based on field collected data under the calculation framework of the HCM2016 [1]. The data was collected by video camera. The saturation flow rate of four movements at the CFI approaches are compared with those at the conventional approaches. The proposed model is validated and the relative error is less than 10%.

The rest of this paper is organized as follows: Section 2 introduced the data collection. The difference in saturation flow rates between the CFI intersections and the conventional intersections was compared and the corresponding saturation flow rate adjustment model was established in Section 3. Section 4 validated the proposed model. Section 5 discussed the recommendations for improvement. Finally, the conclusions were presented.

## Field data preparation

### Measured data

**Fig 2:**
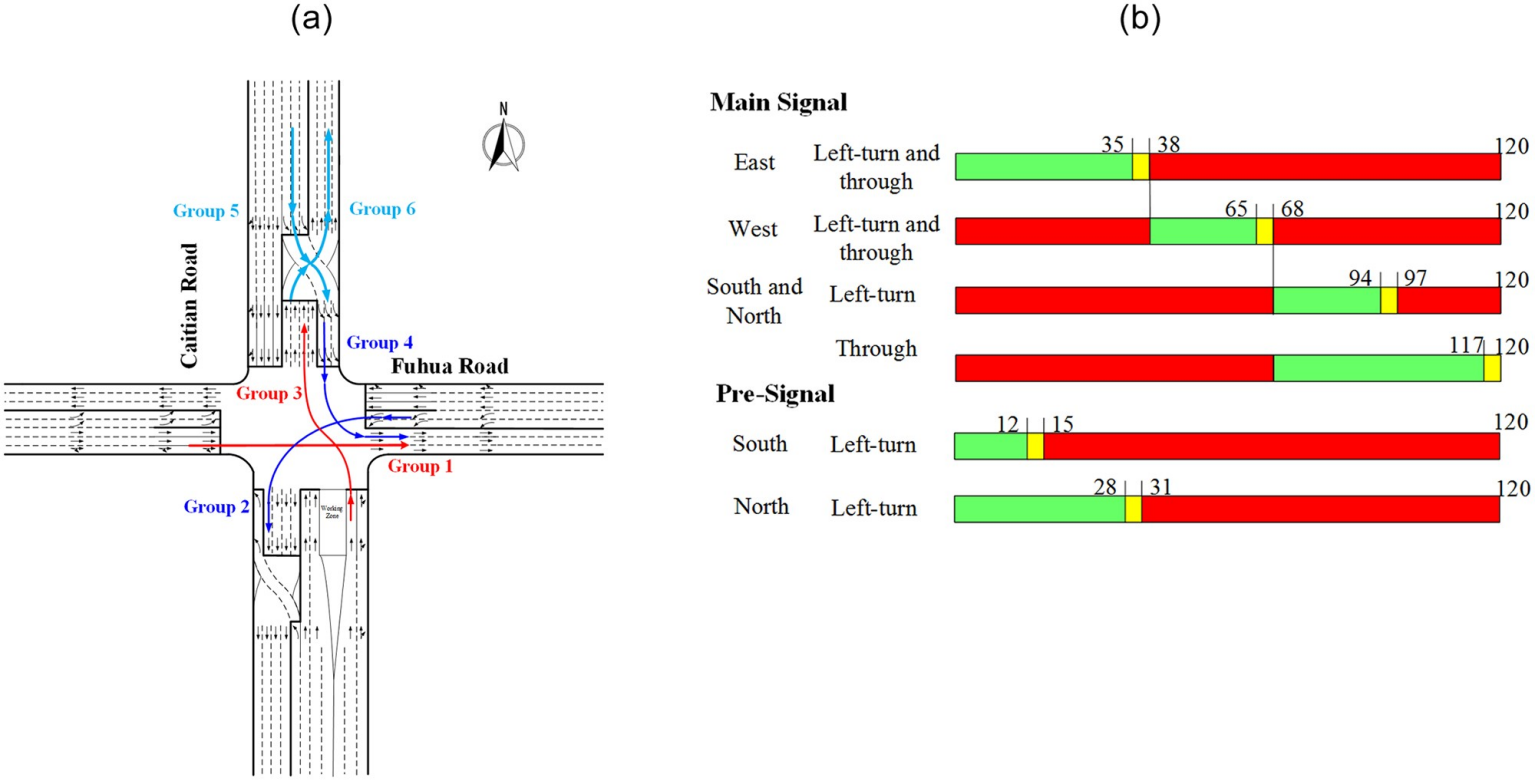
Surveyed intersection. (a) Geometric layout and data grouping. (b) Signal timing.

To obtain the actual operational data, the intersection of Caitian Road—Fuhua Road in Shenzhen, China, was selected as a case for evaluation analysis. This intersection is located in the downtown of Shenzhen. The north and south legs of this intersection have adopted the design of CFI from October 15th, 2017, which is the first CFI built in China. The east and west legs of this intersection are under the conventional design. Its geometric layout and signal timing are illustrated in Fig 2. The intersection information and traffic data were observed and recorded by using video camera located on top of the building nearby during the peak hours [23]. With the video cameras, second-by-second vehicle arrivals and departures status were captured for later analyses [24]. Since it is impossible to get the personal information or vehicular license from the video shooting from the top of vehicles, no identifying information can be obtained. Therefore, such field observations are permitted in China, and no permits are required.

According to HCM2016 [1], the stop line method is generally used for measuring the saturation flow rate at intersections. The saturation flow rate represents the number of vehicles per hour per lane that could pass through a signalized intersection if a green signal was displayed for the full hour, the flow of vehicles never stopped, and there was no large headway. It is computed by Eq (1), on a cycle-by-cycle basis.

$$S=\frac{3600}{h}$$ (1)

where, *S* denotes the saturation flow rate, veh/h; and *h* is the average saturation headway in a signal cycle, s.

Headway is the time between successive vehicles as they pass a point on a lane or roadway, measured from the same point on each vehicle. When the signal turns green, the queue begins to move. The driver of the first vehicle in the queue must observe the signal change to green and react to the change by releasing the brake and accelerating through the intersection. As a result, the first headway will be comparatively long. The third and fourth vehicles follow a similar procedure, each achieving a slightly lower headway than the preceding vehicle. After four vehicles, the effect of the start-up reaction and acceleration has typically dissipated. Successive vehicles then move past the stop line at a more constant headway until the last vehicle in the original queue has passed the stop line. The HCM [1] recommends using the fifth vehicle following the beginning of a green as the starting point for saturation flow measurements. That said, the saturation headway can be obtained by Eq (2). Each time a flow is stopped, it must start again, with the first four vehicles experiencing the start-up reaction and acceleration headways.

$$h=\sum_{i=5}^{l}h_{i}$$ (2)

where, *l* means the last vehicle in queue; *h*_*i*_ means the lost time for *i*^th^ vehicle in queue, s.

The method of video recording was applied to further obtain the average headway of each lane. To ensure that the data are at the sufficient level of accuracy and precision, a specially developed image processing software used to collect the headway has been employed, which has been successfully used in data collection in our previous work [25]. Heavy vehicles and passenger cars were identified by vehicle types in the video according to regulations of Ministry of Public Security of the PRC [26], cars with a length of less than 6 m and fewer than 9 passengers or a total mass less than 4.5 t are small cars. Therefore, trucks, container trucks and buses are subordinated to heavy vehicles while compact cars, minivans belong to passenger cars. After the camera along the road captured the data of every car passing the CFI, the headway of each car can be obtained automatically and then the average headway and the saturation traffic flow of each lane can be calculated.

Both the saturation flow rate of the CFI approach and that of the conventional approach were collected in one intersection because only the north and south legs of this intersection have adopted the CFI design. It can ensure the comparability between different groups because the drivers’ driving behaviours of them can be assumed to be the same. Though there are various factors influencing the saturation flow rate, the emphasis of this paper is on the change of saturation flow rate caused by CFI design and what leads to these changes. Therefore, a special intersection where both the CFI design and the conventional design are simultaneously used was selected to study differences between these two designs. Thanks to such an intersection, the interference of other factors is removed as much as possible. There is no need to consider other influencing factors in HCM.

### Data grouping

In this paper, the differences between the saturation flow rate of CFI approaches and the conventional approaches were analysed by obtaining the correction coefficient. Therefore, according to the different movements, the traffic data were divided into six groups for further comparison and the detailed schematic diagram of each traffic group is illustrated in Fig 2. Besides, there was no variation in signal timing during the survey.

- Group 1 is the through movement at the conventional approach, which is used as a benchmark for through movement.
- Group 2 is the left-turn at the conventional approach, which is used as a benchmark for left-turn.
- Group 3 is the through movement at the main stop-line of CFI.
- Group 4 is the left-turn at the main stop-line of CFI.
- Group 5 is the movement of left-turn vehicles entering the displaced left-turn lanes at the pre-signal of CFI.
- Group 6 is the exit movement entering the road segment at the pre-signal of CFI.

## Modelling

### Statistics description

**Fig 3:**
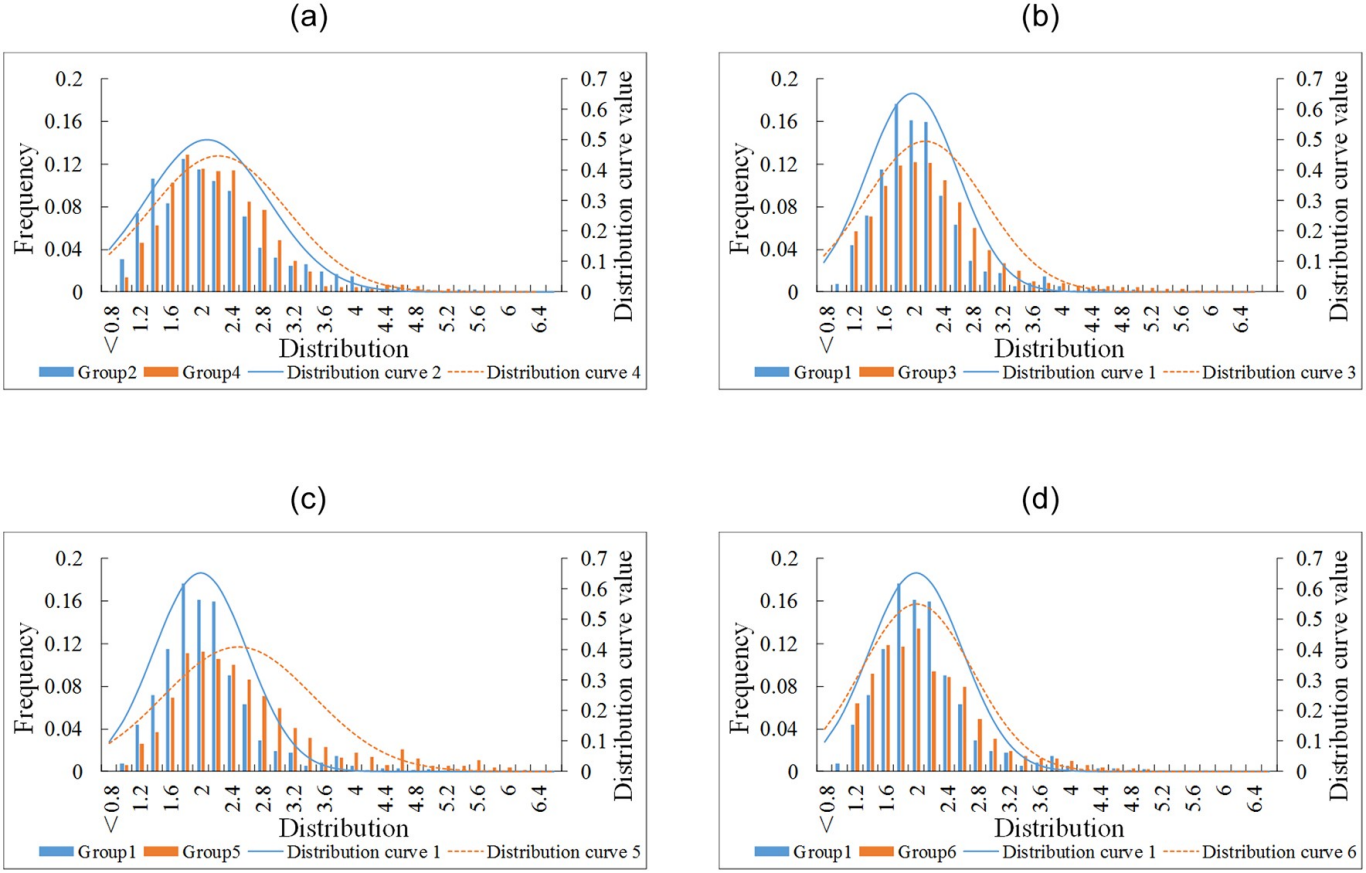
Distribution of saturation headways. (a) Group 4 vs Group 2. (b) Group 3 vs Group 1. (c) Group 5 vs Group 1. (d) Group 6 vs Group 1.

Basic statistical results of saturation headway are shown in Table 1, including the sample size, minimum headway, maximum headway, average headway and standard deviation of headway in each group. To visually demonstrate the difference on the distribution of the saturation headways between the CFI and the conventional design, four comparison pairs were conducted by the histogram, shown in Fig 3.

**Table 1:** Headway statistical description in each group.

	Sample size	Min (s)	Max (s)	Average (s)	Standard deviation
Group1	3194	0.81	5.77	1.992	0.611
Group2	2511	0.82	5.93	2.075	0.799
Group3	5253	0.85	6.28	2.162	0.807
Group4	4573	0.82	6.10	2.227	0.891
Group5	3562	0.88	6.20	2.482	0.977
Group6	5307	0.80	5.11	2.001	0.726

First, the normality of each group data is tested by Kolmogorov-Smirnov test. The result is shown in Table 2, the significance in each group is less than 0.01, which indicates that none of the six groups of data is normally distributed. Therefore, the nonparametric test is applied to analyse each group data’s difference. Based on the analysis of the average rank, the method of Mann-Whitney U is used to test whether the populations of two samples are at the same level. The principle of the Mann-Whitney U test is to get the rank of each data in ascending order and then calculate the average rank of two samples. The calculation results are shown in Table 3. From the last column of the table, it can be statistically said that there are indeed significant differences between each comparison pairs (*Sig* < 0.05) expect for Group 1 vs Group 6. Therefore, the design of the CFI has significant impact on the saturation flow rate. The correction coefficients of the saturate flow rate should be explored.

**Table 2:** Kolmogorov-Smirnov test.

Group	Statistic	Sig
Group1	.056	.000
Group2	.081	.000
Group3	.077	.000
Group4	.052	.000
Group5	.065	.000
Group6	.053	.000

**Table 3:** Significance test for each comparison pairs.

Group pairs	Mann-Whitney U	Wilcoxon W	Z	Sig
Group2 –Group4	4950914.5	8104730.5	-5.764	0.000
Group1 –Group3	7402125	12504540	-9.081	0.000
Group1 –Group5	3863136	8965551	-22.807	0.000
Group1 –Group6	8342447.5	22427225.5	-1.212	0.225

The saturation headways of the left-turn (Group 4) and through movement (Group 3) at the main-signal of the CFI are compared with those at conventional approaches (Groups 2 and 1), respectively, as shown in Fig 3a and 3b, respectively. Moreover, the movements at the pre-signal of CFI were also studied. They can be treated as two opposing through movements. Therefore, the movement that vehicles entering the displaced left-turn lanes (Group 5) and the exit movement entering the road segment at the pre-signal (Group 6) were compared with the Group 1, as shown in Fig 3c and 3d, respectively.

It can be known that the distribution curves of saturation headways at CFI approaches are always more dispersed and closer the right with the comparison of that of the conventional approaches, which indicates the saturation headway and its dispersion degree at CFI approaches are higher. High CFI headway implies less utilisation of traffic lane capacity. Among them, the distribution curve of Group 5 in Fig 3c has obvious difference with that of Group 1, which means the left-turn vehicles entering the displaced left-turn lanes at the pre-signal of CFI were most affected by CFI.

The normality of each group data was tested by the method of Kolmogorov-Smirnov and the results shown the significance of K-S test in each group is less than 0.01, which indicates none of the six sets of data comfort to normal distribution, as shown in Table 2. By comparing the relation distribution curves between the quantile of variable data and specified distribution, it can be seen intuitively that the traffic data of each group are not normally distributed.

Therefore, nonparametric test should be employed to analyse each group data’s difference. Based on the analysis of the average rank, the method of Mann-Whitney U is used to test whether the populations of two samples are the same at the same location. The test principle is to combine two samples and get the rank of each data in ascending order firstly and then calculate the average rank of two samples. The calculation results are shown in Table 3 where there are significant differences between every tested group pairs except for Group 1 and Group 6. Therefore, it can be concluded that the design of the CFI has impact on the saturation flow rate. The correction coefficient of each direction in the saturate flow rate model should be adjusted for CFI, which will be discussed in the following sections.

### Modelling the saturation flow rate adjustment

In this paper, all raw data were divided into two parts. In this section, the correction coefficients of saturation flow rate for different movements at the CFI are established by 80% of the field data. The proposed model will be validated using the left 20% data.

#### Adjustment for left-turn at the CFI approach

**Fig 4:**
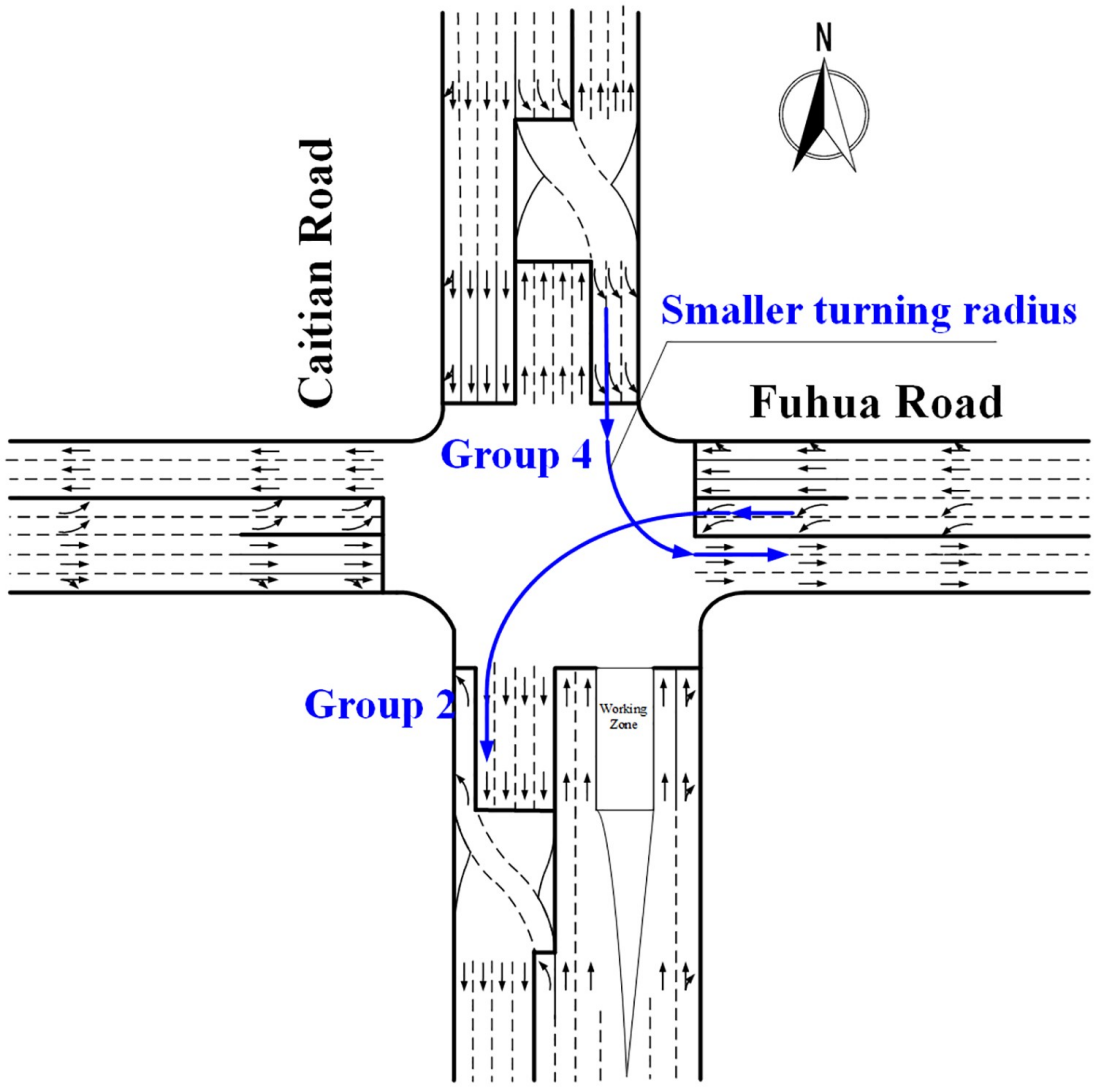
Smaller turning radius of left-turn at the CFI approach.

The construction of CFI generally results in a smaller turning radius, which is the main difference in the operation of the left-turn at the main intersection between CFI and conventional approach, as shown in Fig 4. Since the effect of smaller turning radius on different types of vehicles may be different, the saturation flow rates of passenger cars and heavy vehicles at the CFI need to be discussed separately. The statistical description of passenger cars and heavy vehicles in Groups 2 and 4 are listed in Table 4. As for passenger cars, the average headway is 1.970 and 2.253 respectively at the conventional intersection and the CFI. Moreover, as for heavy vehicles, the average headway is 4.003 and 4.881 respectively at the conventional intersection and CFI.

**Table 4:** Headway descriptive statistics in Group 2 and Group 4.

Group	Sample size	Min (s)	Max (s)	Average (s)	Standard deviation
Passenger cars in Group 2	2381	0.82	4.43	1.970	0.657
Passenger cars in Group 4	1392	0.87	4.52	2.253	0.632
Heavy vehicles in Group 2	130	3.24	5.93	4.003	0.702
Heavy vehicles in Group 4	240	3.25	6.10	4.881	0.752

Therefore, the saturation flow rate of left-turn vehicles at the CFI is 87.4% and 82.0% of the conventional intersection for passenger cars and heavy vehicles, respectively. It indicates the difference in left-turn operating at the CFI of heavy vehicles compared with passenger cars. Thus, the adjustment coefficient of saturation flow rate for left-turn at the CFI approach can be calculated by Eq (3), which indicates that the correction coefficient equals to 0.874 when the proportion of the heavy vehicles is 0%, and correction coefficient equals to 0.820 when the proportion of the heavy vehicles is 100%.

$$f_{CL}=87.4\%-(87.4\%-82.0\%)P_{HV}$$ (3)

where, *f*_*CL*_ is the adjustment coefficient for left-turn at the CFI approach; and *P*_*HV*_ is the proportion of the heavy vehicles.

#### Adjustment for through at the CFI approach

**Fig 5:**
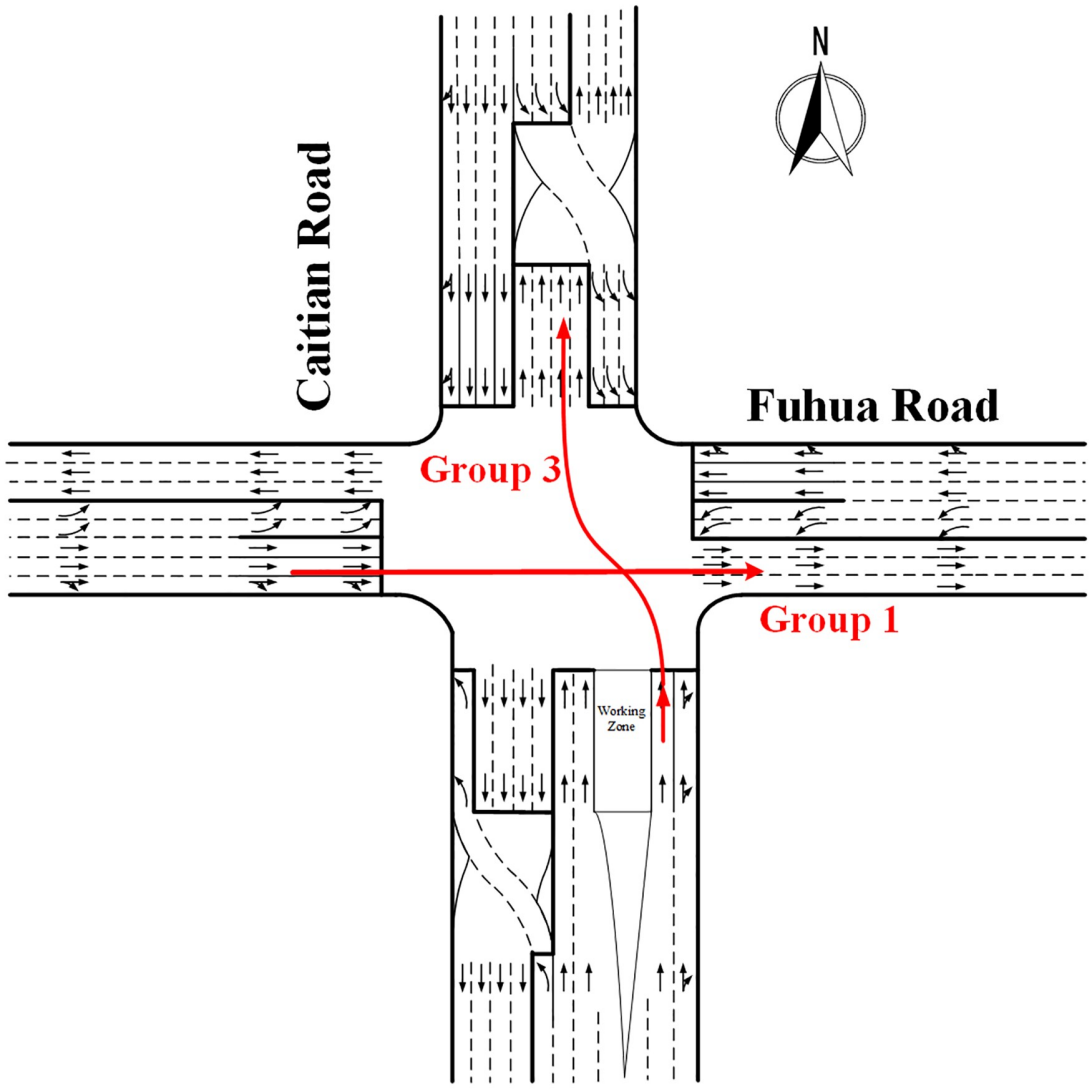
Lane offset of through movement at the CFI approach.

The construction of CFI generally results in the problem of lane offset in the direction of through traffic due to the different number of left-turn lanes, as shown in Fig 5. Therefore, drivers are forced to make lane choices and changing lanes. According to Table 1, the saturation flow rate of Group 3 is 7.86% lower than that of Group 1 on average.

**Fig 6:**
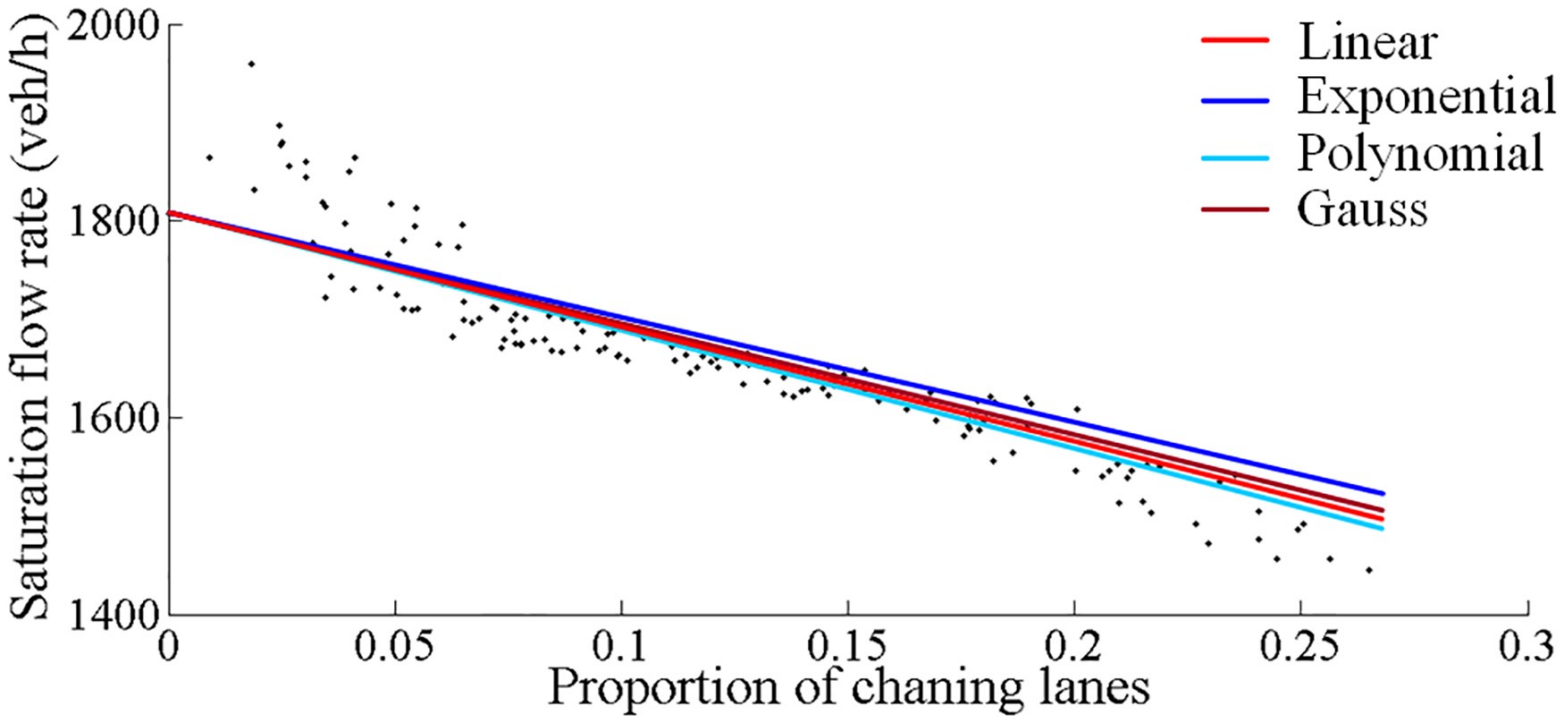
Fitting curves of saturation flow rate at CFI and lane changing proportion.

Table 5 shows the distribution of traffic among the through lanes at the CFI approach and according exit lanes. The utilization ratio of the outermost entrance lane is the lowest while the outermost exit lane is used the most, and their utilization ratios are 0.230 and 0.275, respectively. The saturation flow rate of each cycle and the proportion of traffic volume changing lanes were recorded in detail, as shown in Table 6. The vehicle was regarded as changing lane if it was not on its original corresponding lane when it entered the exit lane. Almost 20% through vehicles did not choose the exit-lane corresponding to the original entrance lane. In turn their behaviour of changing lanes affected the operation efficiency and reduced the saturation traffic flow. The relationship between saturation flow rate and proportion of lane changing can be analysed. Various functions were tried to fit curve, such as linear, exponential, polynomial and Gauss function. The functions with its goodness are listed in Table 7 and fitting curves are shown in Fig 6.

**Table 5:** Lane utilization.

Lane	Entrance lane	Exit lane
Lane 1	0.243	0.226
Lane 2	0.266	0.246
Lane 3	0.261	0.253
Lane 4 (outermost)	0.230	0.275

**Table 6:** Example of the recorded data of lane changing.

Number of cycle	Saturation flow rate	Lane changing proportion
1	1864.802	0.008974
2	1958.780	0.183460
3	1831.900	0.188630
4	1897.019	0.024393
5	1876.591	0.024697
6	1897.699	0.025061
…	…	…
150	1818.182	0.034081

**Table 7:** Functions between saturation flow rate and lane changing proportion.

Function	Fitting result	R-square
Linear	*S*_3_ = −1218*P*_*c*_ + 1814^a^^,^^b^	0.925
Exponential	$S_{3}=1815e^{-0.7585P_{c}}$	0.927
Polynomial	$S_{3}=-920.8P_{c}^{3}-171.5P_{c}^{2}-1209P_{c}+1812$	0.926
Gauss	$S_{3}=2164e^{-{\left(\frac{P_{c}+0.5499}{1.305}\right)}^{2}}$	0.926

^a^*S*_3_ is the saturation flow rate of Group 3.

^b^ *P*_*c*_ is the proportion of lane changing.

Among the tested fitted curves, linear model, exponential model, polynomial model and Gauss model are more appropriate with high goodness of fit. It can be seen that most of scatters are located on these curves and the R-square of all these models is greater than 0.9. Considering the model’s practical application, the simpler the model is, the easier it is to be calculated. Therefore, the concisest model (Linear) was chose to further get the relationship between the adjustment coefficient of saturation flow rate and proportion of lane changing by using the saturation flow rate of Group 1 as a benchmark, and the equation is shown in Eq (4). In Eq (4), the proportion of lane changing vehicles is the independent variable and it should be smaller than maximum value of the field data. It means the applicable range of *P*_*c*_ is from 0 to 0.3.

$$f_{CT}=1-0.709P_{c}\;(0\leq P_{c}\leq 0.3)$$ (4)

where, *f*_*CT*_ is the adjustment coefficient for through movement at the CFI approach; and *P*_*c*_ is the proportion of lane changing vehicles.

#### Adjustment for left-turn at the pre-signal

**Fig 7:**
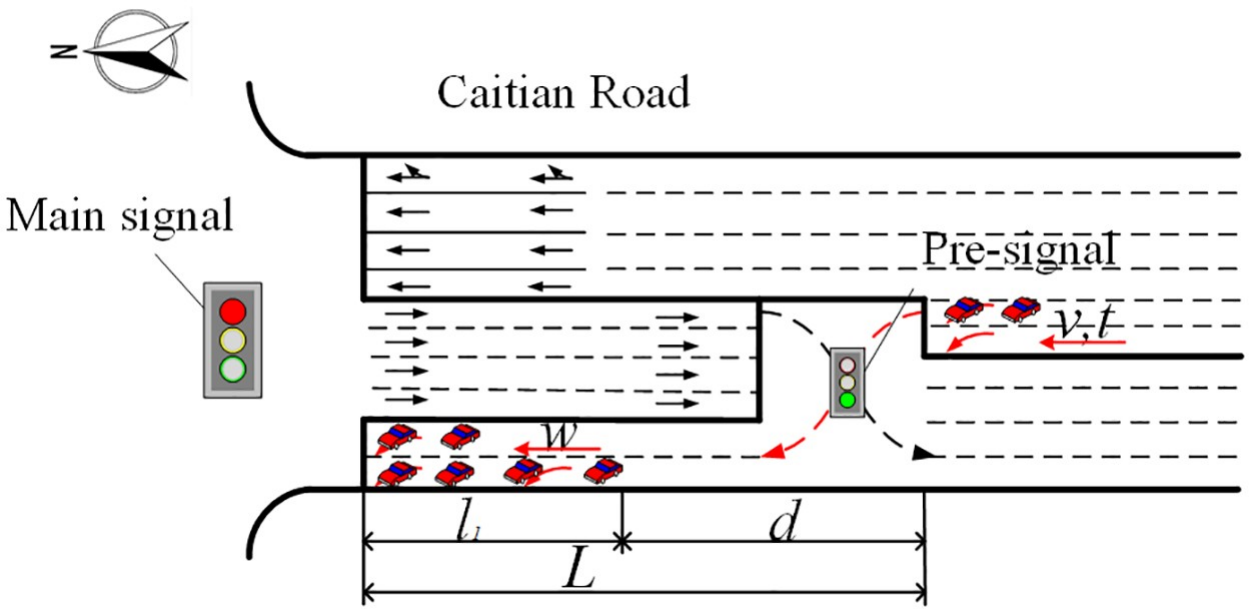
Vehicles entering the displaced left-turn lanes at the pre-signal of CFI.

When the left-turn vehicles enter the displaced left-turn lanes at the pre-signal of CFI (Group 5), these vehicles should queue and wait for the red light of the main-signal, as shown in Fig 7. Therefore, for drivers, it is not necessary to hurry to drive to the main-stop line. According to the field observation, some of the drivers were lazy and lumbering when they entered and passed the displaced left-turn lanes from the pre-stop line to the main-stop line. This phenomenon was more obvious when there were many vehicles queuing in the waiting turn lanes. Therefore, the saturation flow rate was reduced. According to Table 1, the saturation flow rate of Group 5 is 19.74% lower than that of Group 1 on average.

The relationship between the headway and the distance between the end of queue and the pre-stop line (*d*) was analysed. The fitting results are shown in Table 8. Although the Gauss model is the most accurate function fitting to the scatters, the Linear model is more succinct. Therefore, the Linear model was chosen as the function for the relationship between headway of Group 5 and the distance between the end of queue and the pre-stop line (*d*).

$$h_{5}=-0.041d+5.833$$ (5)

where, *h*_5_ is the headway of vehicles enter the displaced left-turn lanes at the pre-signal of CFI; and *d* is the distance between the end of queue and the pre-stop line, m, which can be further determined by the length of displaced left-turn lane and the queue length.

**Table 8:** Regression analysis between headway and *d*.

Function	Fitting result	R-square
Linear	*h*_5_ = −0.041*d* + 5.833 ^a^	0.475
Exponential	*h*_5_ = 9.288*e*^−0.01785*d*^	0.512
Power	*h*_5_ = 51.03*d*^−0.483^ − 3.817	0.513
Rational	$h_{5}=\frac{-1.52d+360.2}{d+22.79}$	0.513

^a^ *S*_3_ is the saturation flow rate of Group 3.

Since the queue length will continue growing from the start of green time of the pre-signal, *d* will change accordingly, which can be specified by Eq (6). Then, the average saturation headway of Group 5 can by calculated by Eq (7).

$$d=\left\{ \begin{array}{cc} L & ,\;0\leq t\leq \frac{L}{v}\\ L-w(t-\frac{L}{v}) & ,\;t>\frac{L}{v} \end{array}\right.$$ (6)

where, *L* is the length between the pre-stop line and the main stop line, m; *t* is time from the start of the green of the pre-signal, s; *w* is the speed of shock wave which can be regarded as 5 m/s [27]; and *v* is the vehicular speed, m/s.

$$\overset{-}{h_{5}}=\frac{\int_{0}^{g}h_{5}dt}{g}$$ (7)

where, *g* is the green time of the phase that left-turn vehicles entering the displaced left-turn at the pre-signal, s.

Substituting Eqs (5) and (6) into Eq (7), the adjustment coefficient of saturation flow rate for left-turn at the pre-signal of CFI can be obtained by using the saturation flow rate of Group 1 as a benchmark, as shown in Eq (8). As the coefficient *f*_*CP*_ is dependent on the length of displaced left-turn lanes (*L*), the applicable range of *L* in Eq (8) can be obtained by the measured length of *d*, which is from 20 m to 120 m.

$$f_{CP}=\frac{h_{1}}{\overset{-}{h_{5}}}=\left\{ \begin{array}{cc} \frac{1.992}{-0.041L+5.833} & ,\;20\leq L<90,g\leq \frac{L}{v}\\ \frac{1.992}{0.1025(g+\frac{L^{2}}{v^{2}g})-0.041(L+\frac{5L}{v})+5.833} & ,90\leq L\leq 120,g>\frac{L}{v} \end{array}\right.$$ (8)

where, *f*_*CP*_ is the adjustment coefficient for left-turn vehicles at the pre-signal of CFI.

#### Adjustment for saturation flow rate for CFI

Considering the above four movements, the saturation flow rate adjustment for CFI can be calculated by Eq (9) along the same line as the saturation flow rate calculation model in HCM2016 [1]. The factor *f*_*CL*_ refers to the adjustment for left-turn at the CFI approach, which can be calculated using Eq (3). The factor *f*_*CT*_ refers to the adjustment for through movement at the CFI approach, which can be calculated using Eq (4). The factor *f*_*CP*_ refers to the adjustment for left-turn at the pre-signal of CFI, which can be calculated using Eq (8).

$$f_{C}=f_{CL}f_{CT}f_{CP}$$ (9)

where, *f*_*C*_ is the adjustment coefficient for CFI.

## Model validation

To validate the accuracy of correction model in each direction, 20% measured data were chosen randomly. The difference between measured saturation flow rate and fitting data calculated by each adjustment function for different movements was compared respectively.

### (1) Validation for left-turn at the CFI approach

The correction coefficient of Group 4 can be calculated by Eq (3) and *P*_*HV*_ is 14.15% in the left 20% data, so the *f*_*CL*_ = 0.874 − 0.054*P*_*HV*_ = 0.866. Then, the adjusted saturation flow rate is 1381 veh/h while the measured saturation flow rate is 1346veh/h. The relative error of this correction model is 2.67%. Therefore, the correction model of saturation flow rate for left-turn at the CFI approach is reasonable.

### (2) Validation for through at the CFI approach

**Fig 8:**
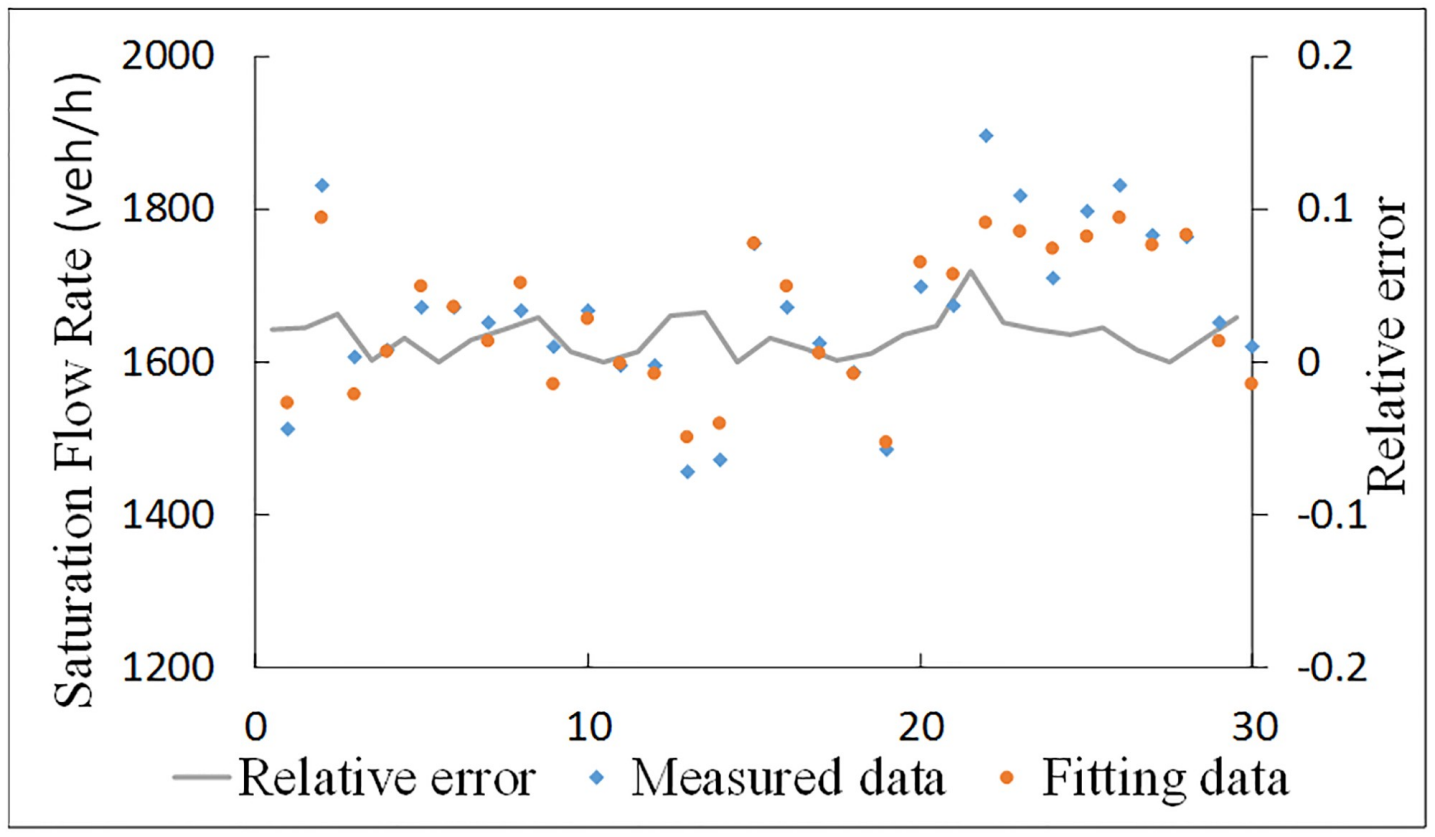
Relative error of correction model for through at the CFI approach.

The correction coefficient of Group 3 can be calculated by Eq (4) in the left 20% data. The accuracy of adjusted saturation flow rate can be validated by the two related samples tests. The results of Wilconxon Signed Ranks Test are shown in Table 9. It shows that the significance of samples is 0.360 (more than 0.05) which means that there is no significant difference between the measured saturation flow rate and calculated saturation flow rate for through movements at CFI. Moreover, the scatters of measured data and fitting data and the relative error are shown in Fig 8. The relative error is always less than 8.0%. Therefore, both the nonparametric test and relative error indicate that the correction model of saturation flow rate for through direction is valid for the actual situation.

**Table 9:** Wilconxon Signed Ranks test for through at the CFI approach.

Statistical items		Values
Negative Ranks	N	18
	Mean Rank	15.389
	Sum of Ranks	227.00
Positive Ranks	N	12
	Mean Rank	15.667
	Sum of Ranks	188.00
Z		-0.915
Asymp Sig(2-tailed)		0.360

### (3) Validation for left-turn at the pre-signal

Since the parameters of *L* and *g* are different at the south and north approach, the correction coefficient for through at the CFI needs to be calculated respectively when testing the validation of the correction model. The actual information about Caitian Road are recorded in the first three columns of Table 10. According to the constraints in the Eq (8), the correction coefficient of south approach should be obtained by the second function while that of north approach should be get by the fourth function. Therefore, the correction coefficients for left-turn at the pre-signal of the south and north approach are 0.635 and 0.846, respectively. Then, the adjusted saturation flow rate in the south and north direction is 1148.093 veh/h and 1529.166 veh/h, respectively. Compared with the measured saturation flow rate, the calculation results of relative error for left-turn vehicles at the pre-signal of CFI are also shown in Table 10. It can be known that the average error for correction model of Group 5 is 8.77%, which shows the accuracy of correction model for left-turn at the pre-signal of CFI approach is acceptable.

**Table 10:** Validation for left-turn at the pre-signal of CFI approach.

Item	Parameter	South approach	North approach	Average
Measured data	Sample Size	1138	2424	
	*L* (m)	70	125	-
	*g* (s)	12	28	-
	Saturation flow rate (veh/h)	1245	1400	1450
Estimated results	*f*_*CP*_	0.635	0.846	-
	Saturation flow rate (veh/h)	1148.093	1529.166	1402.142
Relative error		8.17%	7.78%	9.23%

## Discussion

In this chapter, methods to mitigate the negative impact on the saturation flow rate at the CFI were proposed according to the influencing factors analysed in Section ‘Modelling’.

For the left-turn at the CFI approach, the saturation flow rate of passenger cars and heavy vehicles is 87.4% and 82% respectively, which means the vehicle type affects the traffic flow. Thus, to satisfy different kinds of vehicles and improve the left-turn saturation flow rate at continuous flow intersections, the stop line of the intersecting conventional intersection can be moved backward so that the turning radius will be increased.

For the through movement at the CFI approach, since the lane choice and lane changes caused by lane dislocation is the main reason why the through saturation flow rate of the CFI is less than that of the conventional intersection, the striking coloured guide line can be used and some physical facilities can be added between lanes at the intersection such as yellow flashing lights to streamline the traffic flow and reduce the proportion of lane changing. Moreover, the entrance lanes and exit-lanes can be marked with numbers to guide drivers to drive along its original lane.

For the left-turn at the per-signal, the length of displaced left-turn lanes is a main factor influencing the saturation flow rate. Once the green time of pre-signal is given, it can be determined by Eq (7) under an expected correction coefficient. The recommend values for the length of displaced left-turn lanes under various expected correction coefficient are listed in Table 11.

**Table 11:** Recommended length of displaced left-turn lanes (m).

Green time (s)	Expected adjustment coefficient					
	0.95	0.9	0.85	0.8	0.75	0.7
10	90	85	80	80	75	70
15	95	90	85	85	80	75
20	110	100	95	90	85	80
25	120	115	110	100	95	90
30	135	130	120	115	110	105

## Conclusion

A saturation flow rate adjustment model for CFI is established in this paper. Four movements related with CFI, namely the left-turn at the CFI approach, the through movement at the CFI approach, left-turn at the pre-signal, and exit movement at the pre-signal, are discussed. The proposed model is validated by comparing with the field data. The results show the relative error is less than 8.0%. The methods to mitigate the negative impact on the saturation flow rate at the CFI are also recommended. From the analysis, the following conclusions can be drawn:

1. Overall, the saturation flow rate of the left-turn at the CFI approach, through movement at the CFI approach, and left-turn at the pre-signal are significantly lower than that of the conventional approaches, while exit movement at the pre-signal will not be affected.
2. For the left-turn, through movement, and left-turn at the pre-signal, the degree of the reduction of the saturation flow rate are mainly related to the heavy vehicle ratio (*P*_*HV*_), the proportion of lane changing vehicles (*P*_*c*_), and the comprehensive influence of length of displaced left-turn lanes (*L*), green time (*g*) and vehicular speed (*v*), respectively. These main influencing factors are considered in the proposed saturation flow rate adjustment model.
3. To mitigate the negative impact, the following methods are recommended: moving backward the main-stop line to increase the turning radius, guide line marking and variable message signs can be added to streamline the traffic flow.

In practice, more detailed design of the guidance information, such as traffic signs and lane markings, should be conducted with the consideration of the local driving behaviour to streamline the traffic flow, which can be the direction of the future study.
